# Visualizing Research Trends on Culture Neuroscience (2008–2021): A Bibliometric Analysis

**DOI:** 10.3389/fpsyg.2022.884929

**Published:** 2022-05-06

**Authors:** Han Qing Xu, Chih-Chao Chung, Cheng Yu

**Affiliations:** ^1^College of Science and Technology, Ningbo University, Ningbo, China; ^2^General Research Service Center, National Pingtung University of Science and Technology, Neipu, Taiwan

**Keywords:** cultural neuroscience, bibliometric analysis, evolutionary analysis, research topics, research hotspots

## Abstract

Recently, cultural neuroscience has gained attention as a new, important, and interdisciplinary topic in the field of neuroscience. It helps us understand the interaction of cultural and biological factors over the course of life. This study aims to provide a comprehensive overview of the field to readers and potential researchers engaged in cultural neuroscience research. A bibliometric analysis was performed on 113 articles in the field of cultural neuroscience from 2008 to 2021 using data from the core collection of Web of Science. Network visualization software VOSviewer and ITGInsight were used for performance analysis and science mapping. Specifically, the performance analysis included countries, institutions, authors, papers, and journals, while science mapping analyzed the collaboration network, keyword network, bibliographic coupling network, and time series evolution. The results showed that the United States was the most productive country, Northwestern University was the most influential research institution, Chiao Jy was the most influential scholar, and “Social Cognitive and Affective Neuroscience” made the greatest contribution to publishing in the field of cultural neuroscience. Furthermore, collaboration is expected to be the development trend in the future. The key research topics in the field of cultural neuroscience included neuroimaging and psychiatric diseases, theoretical methods, interdisciplinary research, cultural differences (collectivism and individualism), and brain functions. Finally, future research will focus on cultural neuroscience, culture, and self, while adolescence will be the emerging research frontier.

## Introduction

Neuroscience has become increasingly important in academia (Yeung et al., 2017). According to the latest search results, the core collection of the Web of Science (WOS) database contains 42,559 published literature on neuroscience. Related research in this field has been conducted worldwide. In the early stages, research on neuroscience was more focused on natural science fields, such as brain biology (Vaiana and Muldoon, 2020) and medicine (Nguyen et al., 2020). However, with the development of the field, many scholars started paying attention to the application of neuroscience methods in social sciences, integrating external environmental factors, such as culture to enhance the application value of neuroscience.

Recently, cultural neuroscience has emerged as an interdisciplinary branch in the field of neuroscience (Han et al., 2013), involving a wide range of disciplines, such as anthropology, cultural psychology, neuroscience, and neurogenetics. Since its birth, cultural neuroscience has aroused a wave of enthusiasm in academia, and research has also rapidly progressed on cultural neuroscience from diverse perspectives. While some of these studies have focused on theoretical methods and standard frameworks (Chiao et al., 2020; Kwon et al., 2021), others have analyzed the practical application of cultural neuroscience methods (Hamada, 2018; Chen and Qu, 2021). In addition, these studies and academic achievements have combined the theoretical methods of cultural neuroscience with many disciplines to derive new research themes. However, to the best of our knowledge, there are few studies on effectively sorting out the results of research on cultural neuroscience.

Bibliometric analysis is a popular and rigorous method for exploring and analyzing large amounts of scientific data, enabling us to quickly understand the evolutionary characteristics of a particular field while highlighting emerging themes (Donthu et al., 2021a). The bibliometric analysis method first appeared in the field of library and information science (LIS), which used quantitative statistical analysis to conduct a comprehensive review of existing literature (Broadus, 1987). Bibliometric analysis has been used in many disciplines to date, including psychology. For example, Khan et al. (2021) conducted a quantitative analysis of articles on tourism and hospitality management strategies in the post-pandemic era. The results showed that relevant management strategies play an important role in helping managers respond to the survival and development of the post-crisis hotel and tourism industry. Novo et al. (2021) conducted a bibliometric analysis of international scientific literature in the field of art therapy and found that research themes focused on psychology and rehabilitation. Luo et al. (2021) used the scientific knowledge graph tool Citespace to conduct a bibliometric analysis of scientific literature under the theme of pain catastrophizing to explore the research hotspots and frontiers in this field. Bibliometric articles related to neuroscience topics were also common (Alhibshi et al., 2020; Issac and Issac, 2020; Yan et al., 2021). The bibliometric analysis method is suitable for the current research because its quantitative nature limits author bias (Donthu et al., 2021b). Simultaneously, it can process a large amount of data and provide insights into the knowledge structure characteristics and developmental trends of the research subject. Therefore, this study used bibliometric analysis to conduct a comprehensive review of the field of cultural neuroscience. Specifically, the research questions were as follows:

Question 1: Which countries, regions, institutions, and scholars are the most productive and influential in the field of cultural neuroscience?

Question 2: What is the model of collaboration in the field of cultural neuroscience?

Question 3: What are the core themes and evolution trends of cultural neuroscience research?

Question 4: What are the recent research trends in cultural neuroscience?

This paper is organized as follows. The first section briefly reviews the field of cultural neuroscience. The second section describes the research data and methods, while the third section uses the network visualization software VOSviewer (van Eck and Waltman, 2010) and ITGInsight (Wang et al., 2021) to conduct a performance, collaboration network, keyword network, bibliographic coupling network, and research frontier analyses. The fourth section summarizes the findings of the study. A statistical analysis and content mining of relevant literature in the field of cultural neuroscience are conducted using bibliometric analysis methods and network visualization software, based on the core data set of Web of Science. The important publications and most influential countries, institutions, and authors in the field of cultural neuroscience are summarized. The collaboration network, research hotspots, and research frontiers of cultural neuroscience, as well as the evolution path and development trend are analyzed. Finally, the contributions to the field of cultural neuroscience are discussed.

## Research Data and Methods

### Data Collection

The literature included in the two citation databases of SCIE and SSCI in the core set of Web of Science were selected as the data sources. The retrieval topic TS = “Cultural Neuroscience” retrieved 124 records on December 31, 2021, with the retrieval standard selected from January 1, 2008, to December 31, 2021. The “document type” was limited to “article,” the source type was limited to “Journal.” A total of 113 related articles were obtained after removing nine invalid articles. The document type was limited to “article” because journal articles represented the most impactful research works (Marques and Franco, 2020), and simultaneous use of all document types would make it “challenging and costly” to analyze and interpret the findings (Hosseini et al., 2018). This was the basic data source for conducting the current research.

### Data Cleaning

In previous studies, few scholars cleaned the collected data set, which caused several errors when conducting performance analysis on indicators, such as authors, regions, countries, and keywords. Therefore, the knowledge unit to be analyzed needed to be cleaned before the formal analysis. Compared to studies in which only the keywords were cleaned, this study performs data cleaning successively for all knowledge units, including countries, institutions, authors, and keywords. According to the data cleaning rules proposed by Lozano et al. (2019), the transformations are carried out by removing spelling errors, combining singular and plural, removing hyphens, merging synonyms and acronyms, and so on. The final cleaning rules are shown in Table 1.

**Table 1:** Data cleaning rules.

Cleaning field	Cleaning rules	Raw data	Data after cleaning
Institution	Merge synonyms	Aarhus Univ	Aarhus univ
		Univ Aarhus	
		Kaohsiung Med Univ Hosp	Kaohsiung med univ
		Kaohsiung Med Univ	
Keyword	Combine singular and plural	Mental Disorders	Mental disorder
		Mental Disorder	
		Human	Human
		HUMANS	
		Humanism	
	Merge acronyms	CN	Cultural neuroscience
	Removing hyphens	Self-construal	Self-construal

*Only the institution and keyword need to be cleaned, other analysis fields are checked without exception, such that they do not appear in the table*.

### Science Mapping

The scientific map drawing was divided into two parts. First, the network visualization software VOSviewer was used to draw the collaboration network, keyword network, and bibliographic coupling network analyses. In the process of scientific research cooperation, researchers use the knowledge and expertise of collaborators to complete specific research (Fatt et al., 2010). Thus, collaboration network analysis helps us select potential research partners for successful innovation and publishing of future research. Keyword networks are used to analyze research knowledge topics and hotspots in disciplines (Wang et al., 2018; Bai et al., 2021). This was used here to analyze the characteristics of knowledge structure in cultural neuroscience. The Louvain algorithm was applied to bibliographic coupling network analysis to dynamically examine the inflow and outflow of knowledge community nodes (Papers) and the changes in the association between nodes (papers) using a huge amount of data (Blondel et al., 2008). This is beneficial for the classification of knowledge communities in cultural neuroscience. Second, the network visualization software ITGInsight was used to perform theme evolution mapping and discover the latest research trends in cultural neuroscience.

## Results

### Performance Analysis

#### Annual Publications Analysis

**Figure 1:**
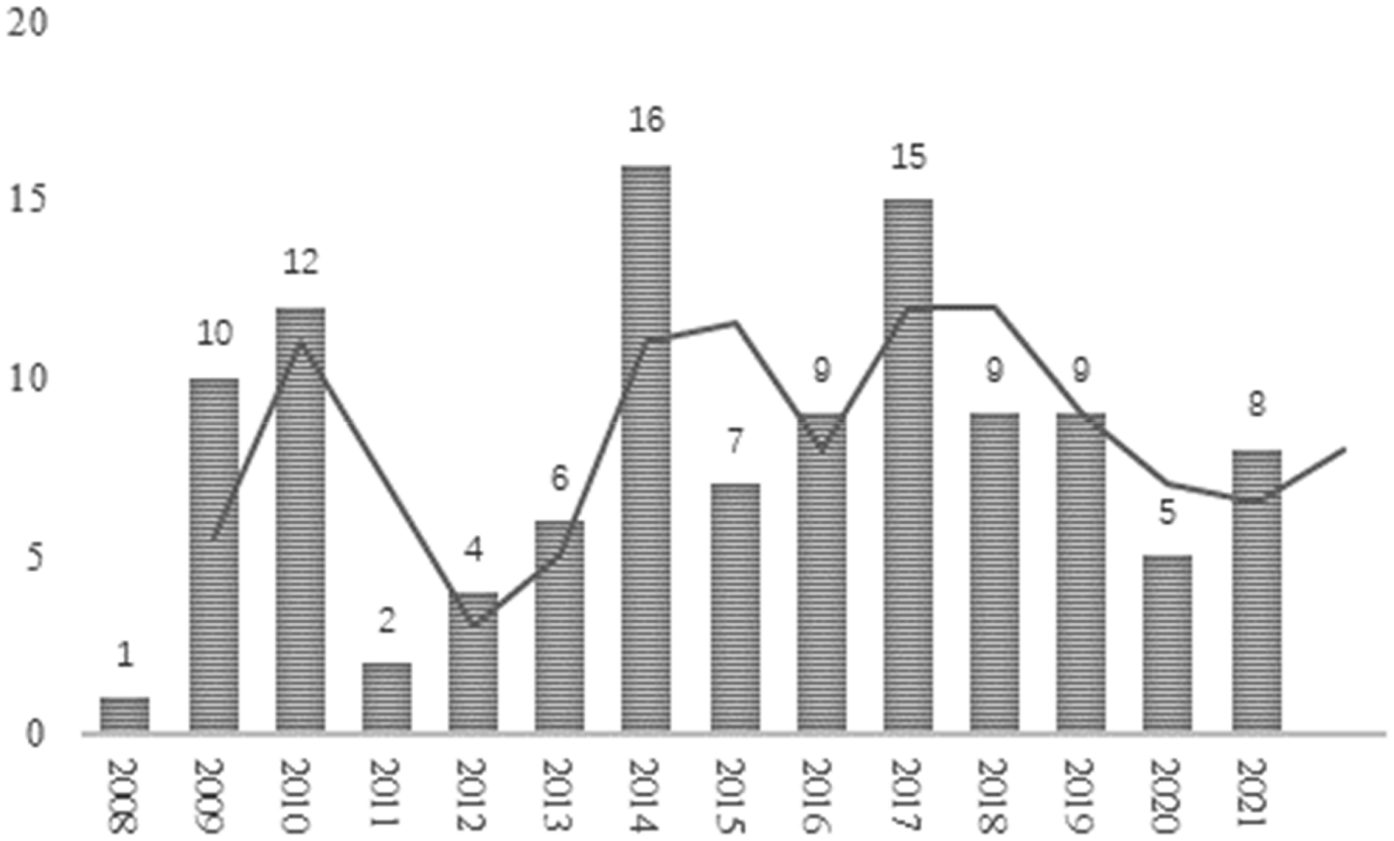
Trends in publications in the field of cultural neuroscience.

The change in the time series of the number of academic papers is an important indicator of a field’s development. A natural year can be taken as a time window to observe the changes in the number of publications, showing the level of academic attention to cultural neuroscience. According to statistics in Figure 1, the earliest scientific paper related to cultural neuroscience was an article entitled “Investigation and validation of intersite fMRI studies using the same Imaging hardware” published in the “Journal of Magnetic Resonance Imaging” in 2008. The article introduced the use of fMRI technique, tested four subjects at different regional sites (Asia, United States), and found that differences between cultural groups should not be attributed to systematic intersite variability, but rather to population systemic differences in interneuron function. This analysis laid the foundation for cross-cultural quantitative analysis of geographically separated populations (Sutton et al., 2008). The most cited article in the field of cultural neuroscience was the article published in “Proceedings of the Royal Society B: Biological Sciences” in 2010. This paper studied the association between culture–genes and concluded that this culture–gene association might explain global variability in the prevalence of pathogens and affective disorders (Chiao and Blizinsky, 2010).

The development of the field of cultural neuroscience can be roughly divided into two stages: The embryonic stage (2008–2010) was characterized by a rapid change in the number of published papers. During this period, the published papers mainly focused on the exploration of theories and methods of cultural neuroscience. In the development stage (2011–2021), research focused on the practical application of many disciplines. However, the volatility of the number of publications also implied that the development of cultural neuroscience during this period was unstable. Although cultural neuroscience has attracted a considerable amount of attention in academia, existing limitations affected its rapid development. Reading the literature suggests that there may be two main reasons for this limitation: First, cultural neuroscience research often requires the technical tool fMRI, but the use of functional magnetic resonance imaging(fMRI) technology is both costly and time-consuming (Sutton et al., 2008). Second, cultural neuroscience is an emerging discipline. Hence, only a few clinicians, researchers, or educators are aware of the field and utilize it in their daily research work (Bhui, 2018).

#### The Most Influential Countries

A total of 27 countries are involved in research in the field of cultural neuroscience. Table 2 lists the top 20 most influential countries. The United States boast of the largest contribution in the field of cultural neuroscience, with 58 published articles and 1,844 citations. Germany had the second largest number of publications, with 18 published papers and 560 citations, followed by the Peoples Republic of China, with 14 published papers published and 523 citations. These contributions to cultural neuroscience imply that cultural neuroscience has emerged as a global, extensive, and diverse research topic.

**Table 2:** Top 20 most influential countries/regions.

Rank	Country	TP	TC	TC|P
1	United States	58	1844	31.79
2	Germany	18	560	31.11
3	China	14	523	37.36
4	England	13	424	32.62
5	Canada	11	449	40.82
6	Australia	7	139	19.86
7	Japan	6	90	15.00
8	Denmark	5	225	45.00
9	Russia	5	38	7.60
10	South Korea	4	181	45.25
11	Netherlands	4	129	32.25
12	Singapore	3	125	41.67
13	Wales	3	58	19.33
14	Switzerland	3	36	12.00
15	Taiwan	3	2	0.67
16	Argentina	2	26	13.00
17	Austria	2	23	11.50
18	Iran	2	9	4.50
19	Uganda	1	26	26.00
20	France	1	23	23.00

*TP represents all papers published in a country/region, TC represents the total number of citations for all papers in a country/region, and TC/P represents the average number of citations for all papers in a country/region*.

#### The Most Influential Institutions

Table 3 lists the top 20 institutions with the highest number of publications. The top three core research institutions were northwestern university with 15 publications, 655 total citations, and 43.67 average citations. The University of Michigan published 12 papers, with 529 total citations, and 44.08 average citations, and Peking University published nine papers, with 437 total citations, and 48.56 average citations. Additionally, Cologne University and Yale University were the top 2 institutions in terms of average citations, reaching 109.50 and 107.00, respectively. Northwestern University, University of Michigan, and Yale University are in the United States, Peking University is in China, and Cologne University is in Germany. These results reveal that different regions (Asia, Europe, and America) have the potential to achieve significant results in cultural neuroscience research, confirming the promising future of research in the field.

**Table 3:** Top 20 most influential institutions.

Rank	Institution	TP	TC	TC/P
1	Northwestern University	15	655	43.67
2	University of Michigan	12	529	44.08
3	Peking University	9	437	48.56
4	London’s Global University	5	117	23.40
5	Aarhus University	4	207	51.75
6	King’s College London	4	166	41.50
7	University of California Los Angeles	4	38	9.50
8	Arizona State University	4	17	4.25
9	McGill University	3	132	44.00
10	Stanford University	3	89	29.67
11	Melbourne University	3	74	24.67
12	Cardiff University	3	58	19.33
13	Marburg University	3	45	15.00
14	Chinese Acad Sci	3	41	13.67
15	Cologne University	2	219	109.50
16	Yale University	2	214	107.00
17	Ottawa University	2	178	89.00
18	Harvard University	2	133	66.50
19	University of California, Santa Barbara	2	97	48.50
20	York University	2	97	48.50

*TP represents the total number of papers published by an institution, TC represents the total number of citations for all papers in an institution, and TC/P represents the number of citations for all articles in an institution*.

#### The Most Influential Authors

Table 4 presents the most influential researchers in the field of cultural neuroscience. Among the top 20 most influential authors, the lowest average citation was 34.75 while the highest was 149. A total of 16 scholars have been cited ≥50 times. Among them, Chiao had the most published papers and the highest number of citations in the field of cultural neuroscience. He has produced 12 papers with 607 citations. Chiao’s early research focused on theories and methods in cultural neuroscience (Chiao and Blizinsky, 2010; Chiao et al., 2010). However, his research focus has recently shifted to the application of cultural neuroscience methods and theories in global mental health (Chiao, 2018; Chiao et al., 2020). Next, both Kitayama and Han produced eight papers and gained 492 and 427 citations, respectively. Kitayama studied the correlation between culture and brain function, focusing more on constructing the role of culture in brain functions from a theoretical perspective (Kitayama and Park, 2010; Kitayama and Salvador, 2017). Han’s research focused on the use of fMRI technology in studying the neural mechanisms related to self-difference in diverse cultures (Han et al., 2013; Ma et al., 2014a; Shi et al., 2016).

**Table 4:** Top 20 most influential authors.

Author	H-index	G-index	TP	TC	YS
Chiao Jy	8	12	12	607	2009
Kitayama S	8	8	8	492	2009
Han S	7	8	8	427	2009
Blizinsky Kd	2	2	2	298	2010
Northoff G	3	3	3	223	2009
Vogeley K	2	2	2	219	2009
Park J	3	3	3	211	2010
Roepstorff A	3	3	3	206	2009
Varnum Mew	3	5	5	177	2013
Harada T	3	3	3	174	2010
Wexler Be	1	1	1	160	2013
Parrish Tb	3	3	3	152	2010
Ma Y	4	4	4	139	2012
Kirmayer Lj	3	3	3	132	2009
Borsboom D	1	1	1	126	2019
Cramer Aoj	1	1	1	126	2019
Kalis A	1	1	1	126	2019
Callard F	1	1	1	108	2015
Fitzgerald D	1	1	1	108	2015
Kim Hs	2	2	2	97	2014

*H-index implies that a scholar has N cited papers for at least N times in all his academic articles; G-index refers to a scholar’s first g papers (sorted according to the number of citations) that are not cited less than g squared papers; TP represents the number of all papers published by an author; TC represents the total number of citations of an author’s papers; and YS represents the time point when an author first published a paper*.

#### The Most Influential Articles

Although only 113 papers were published in the field of cultural neuroscience, the h-index was 32 (at least 32 papers, each with at least 32 citations). Moreover, articles related to cultural neuroscience have easily attracted the attention of the academic community. Table 5 lists the 20 most influential papers.

**Table 5:** The 20 most influential papers.

Rank	Title	TC	Year
1	Culture-gene coevolution of individualism–collectivism and the serotonin transporter gene	282	2010
2	A cultural neuroscience approach to the biosocial nature of the human brain	160	2013
3	Brain disorders? not really: why network structures block reductionism in psychopathology research	126	2019
4	Cultural neuroscience of the self: understanding the social grounding of the brain	122	2010
5	Social science and neuroscience beyond interdisciplinarity: experimental entanglements	108	2015
6	Cultural neuroscience: a once and future discipline	99	2009
7	Cultural influences on neural basis of intergroup empathy	95	2011
8	Enculturing brains through patterned practices	91	2010
9	Cultural neuroscience: biology of the mind in cultural contexts	81	2014
10	Cognitive style as environmentally sensitive individual differences in cognition: a modern synthesis and applications in education, business, and management	75	2014
11	What kind of science for psychiatry?	72	2014
12	How culture gets embrained: cultural differences in event-related potentials of social norm violations	65	2015
13	The brain–artefact interface (bai): a challenge for archaeology and cultural neuroscience	63	2010
14	Neural representations of close others in collectivistic brains	63	2012
15	Contextualizing culture and social cognition	59	2009
16	Novelty-seeking drd4 polymorphisms are associated with human migration distance out-of-africa after controlling for neutral population gene structure	58	2011
17	Culturing the adolescent brain: what can neuroscience learn from anthropology?	56	2010
18	Sociocultural patterning of neural activity during self-reflection	56	2014
19	adhd and the drd4 exon iii 7-repeat polymorphism: an international meta-analysis	54	2010
20	Error-related brain activity reveals self-centric motivation: culture matters	48	2014

“Culture–gene coevolution of individualism–collectivism and the serotonin transporter gene” ranked first and was authored by Chiao and Blizinsky (2010). This paper suggests that human behavior is influenced by both culture and gene expression, which was experimentally tested. The theory of culture–gene coevolution is expected to play an important role in future cultural neuroscience research. This was followed by “A cultural neuroscience approach to the biosocial nature of the human brain” written by Han et al. (2013), providing an overview of the origins, goals, methods, and future development of cultural neuroscience. “Brain disorders? not really: why network structures block reductionism in psychopathology research” written by Borsboom et al. (2019) ranked third among the top-cited papers; it proposed that the use of network models could analyze mental disorders, which was contrary to the finding that cultural and historical context largely affected the strength of network relationships. Therefore, this research was necessary for the advancement of mental disorders research. The fourth most cited paper was “Cultural neuroscience of the self: understanding the social grounding of the brain” written by Kitayama and Park (2010), which explains that the interdisciplinary field of cultural neuroscience investigates interrelations among culture, mind, and the brain, and discusses the impact of culture on brain research. The fifth most cited article was “Social science and neuroscience beyond interdisciplinarity: experimental entanglements” authored by Fitzgerald and Callard (2015). This article describes the dynamics of interaction across the fields of social sciences and neurosciences, through an in-depth analysis of the relationship between social culture and neurobiological knowledge, thus facilitating better cooperation between social scientists and neuroscientists.

#### The Most Influential Journals

A total of 65 journals have published papers on cultural neuroscience. Table 6 lists the 20 most influential journals. Among them, the journal “Social cognitive and affective neuroscience” has made the greatest contribution to the field of cultural neuroscience, with 18 published articles, 653 citations, and 36.28 average citations. The journal “Cultural neuroscience: cultural influences on brain function” ranked second, with eight published articles related to cultural neuroscience, 653 citations, and 36.28 average citations. This was followed by “Frontiers in human neuroscience,” publishing eight related articles, with 163 citations and 20.39 average citations. The journal with the highest average citations was “Proceedings of the royal society b-biological sciences.” Although only one related paper was published in this journal, 282 citations were obtained. An interesting phenomenon was found in that most of these representative journals were interdisciplinary journals—that is, the journals belonged to multiple disciplinary categories. For example, “Social cognitive and affective neuroscience” involved three disciplines: neuroscience, psychology, and experimental disciples; “Frontiers in human neuroscience” involved the disciplines of psychology and neuroscience; “Neuroimage” involved four disciplines, including neuroscience, neuroimaging, radiology, and nuclear medicine and medical. Thus, interdisciplinary integration has become a primary research direction of cultural neuroscience.

**Table 6:** Top 20 most influential journals.

Rank	Journal	TP	TC	TC|P
1	Social cognitive and affective neuroscience	18	653	36.28
2	Cultural neuroscience: cultural influences on brain function	8	253	31.63
3	Frontiers in human neuroscience	8	163	20.38
4	Social neuroscience	6	99	16.50
5	Neuroimage	4	115	28.75
6	Biological psychology	3	10	3.33
7	Annual review of psychology	2	241	120.50
8	Cortex	2	50	25.00
9	Perspectives on psychological science	2	29	14.50
10	Neuroscience and biobehavioral reviews	2	20	10.00
11	Emotion review	2	15	7.50
12	Cultural diversity and ethnic minority psychology	2	5	2.50
13	Sotsiologicheskie issledovaniya	2	5	2.50
14	Integrative psychological and behavioral science	2	3	1.50
15	Proceedings of the royal society b-biological sciences	1	282	282.00
16	Behavioral and brain sciences	1	126	126.00
17	Theory culture & society	1	108	108.00
18	Neural networks	1	91	91.00
19	Psychological science in the public interest	1	75	75.00
20	Trends in cognitive sciences	1	59	59.00

*TP represents the total number of papers published in a journal, TC represents the total number of citations for all papers in a journal, TC/P represents the average number of citations for all articles in a journal*.

### Collaboration Network Analysis

Collaboration network analysis is crucial for understanding academic exchanges and knowledge diffusion. Three types of collaboration networks are commonly used in collaboration analysis research: country collaboration networks (macro), institution collaboration networks (meso), and author collaboration networks (micro). This study separately analyzes these collaboration networks to explore the characteristics of disciplinary knowledge exchange and collaboration patterns in the field of cultural neuroscience.

#### Country Collaboration Network Analysis

**Figure 2:**
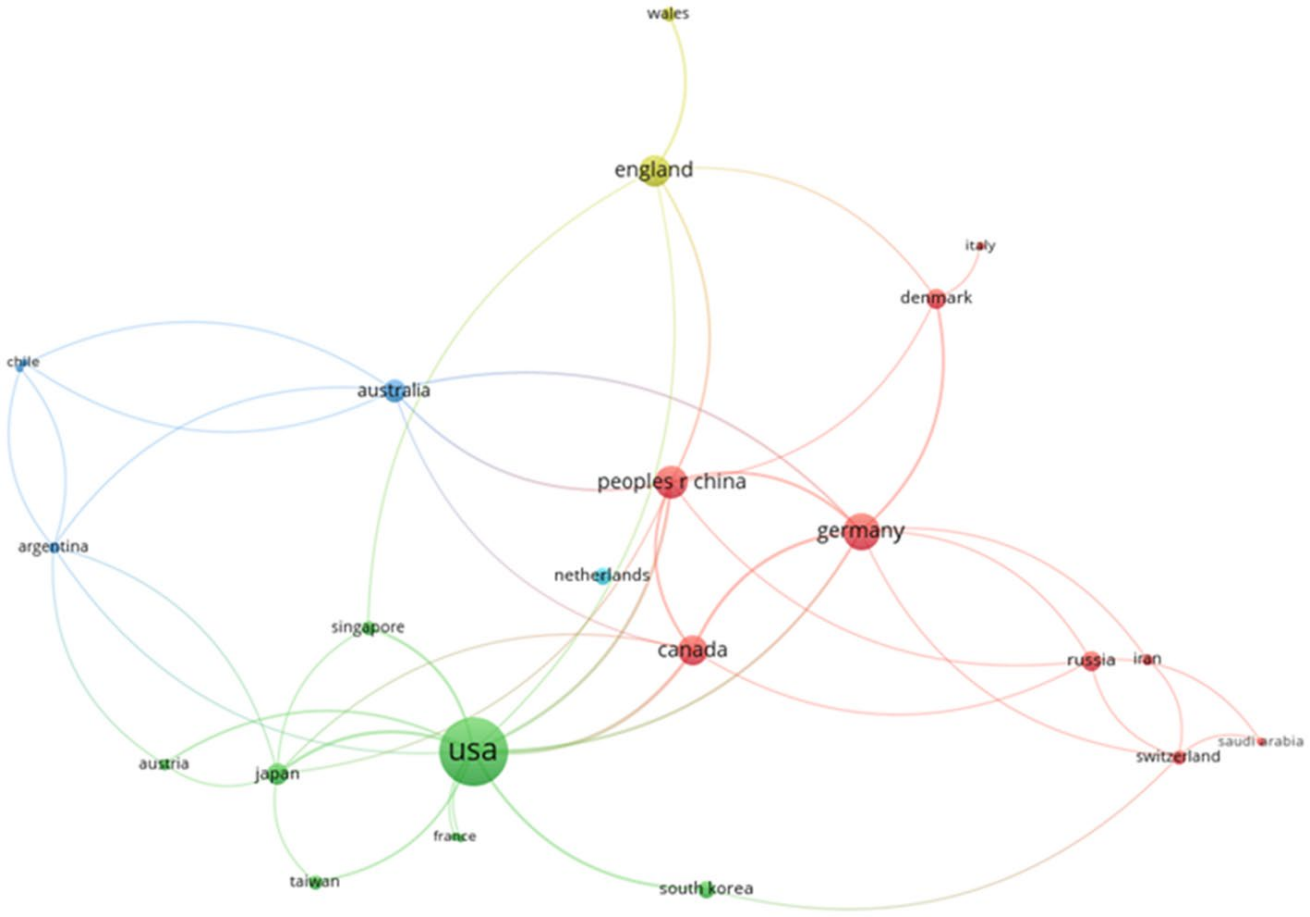
Country collaboration network.

With countries as nodes and the collaboration relationship between countries as the connection, a country collaboration network was created in the field of cultural neuroscience as shown in Figure 2. It contains a total of 27 nodes, with each node representing a country and the line indicating the existence of a collaboration relationship between countries; the thicker the line, the closer the collaboration relationship. The size of the circle reflected the number of papers in a country, and the larger the number of papers, the bigger the circle. A total of 23 nodes (countries) in 27 countries formed the largest connected branch, showing that the four countries of Bulgaria, Netherlands, Poland, and Turkey have no collaboration relationship with other countries. The largest network components also represented major contributors to the field of cultural neuroscience. Researchers usually extract the largest connected branch and use its network structure characteristics to analyze the characteristics of the overall collaboration network (Gonzalez-Alcaide et al., 2015). The largest connected branch in the figure contained the three countries with the highest centrality: The United States (degree = 12), Germany (degree = 12), and China (degree = 12). These core countries have cooperated more than three times. The research trend in the field is represented by the national collaboration in different regions: the collaboration between high-yield countries/regions was also more frequent, with the emergence of the phenomenon of rich clubs (McAuley et al., 2007).

#### Institution Collaboration Network Analysis

**Figure 3:**
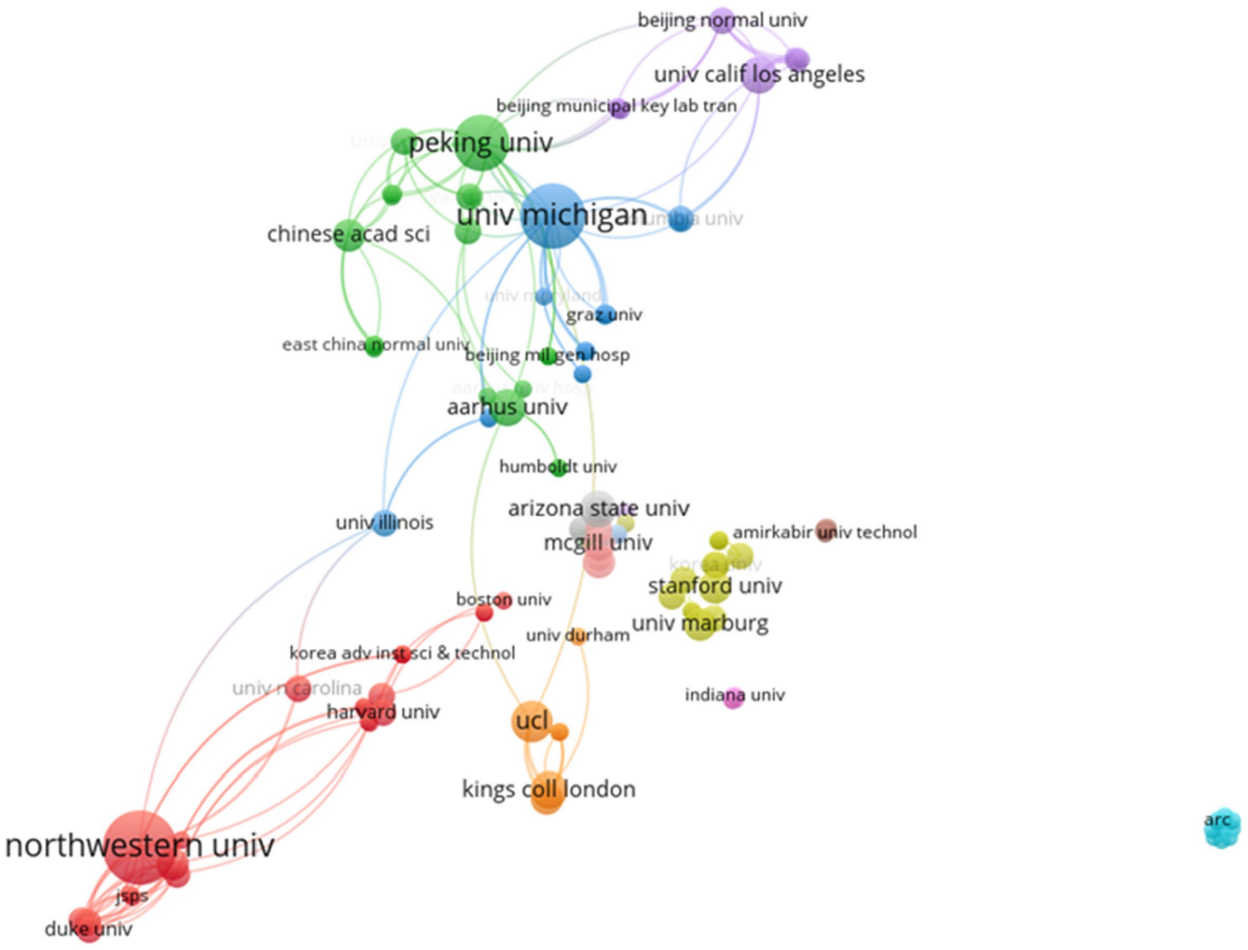
Institution collaboration network.

Figure 3 shows that 116 institutions are involved in the field of cultural neuroscience. The institution collaboration network of cultural neuroscience is composed of a large connected branch and individually existing nodes (institutions). Among them, 77 institutions formed the largest connected branch, accounting for more than half of the research institutions, and 20 research institutions did not cooperate with other institutions. The three research institutions with the highest degree of centrality were the University of Michigan (degree = 23), Peking University (degree = 22), and Northwestern University (degree = 13). These three research institutions formed the core position in the field of cultural neuroscience, with the University of Michigan (United States) having a close collaboration with Peking University (China) and Northwestern University forming a closer collaboration with local research institutions in the United States. This phenomenon reflects the diversity of collaboration among institutions.

#### Author Collaboration Network Analysis

**Figure 4:**
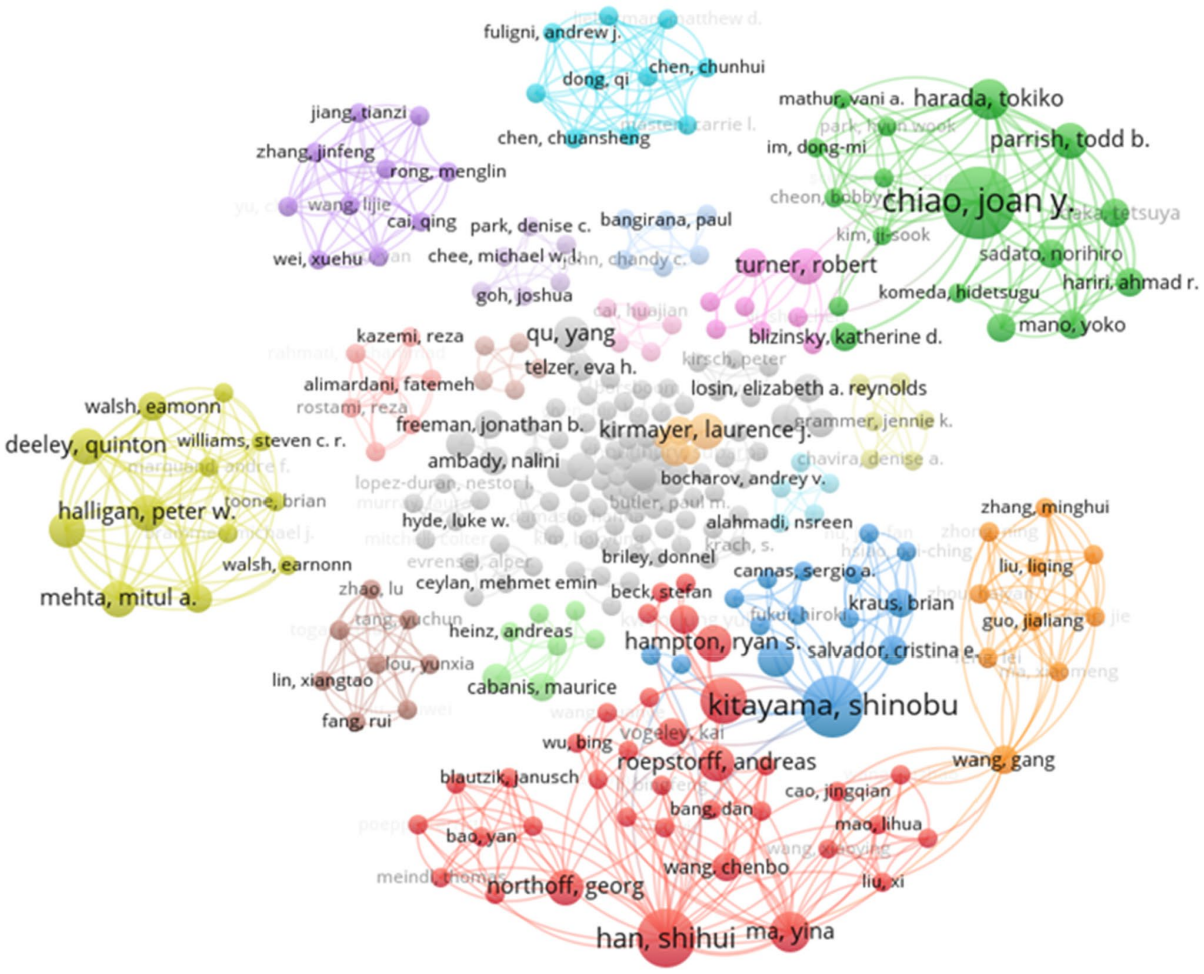
Author collaboration network.

Figure 4 shows that 292 scholars participated in research on cultural neuroscience. Similar to most scientific research collaboration networks, the author collaboration network in the field of cultural neuroscience is fragmented and composed of multiple connected branches. The largest branch in the figure contains 61 authors, and the number of single-author branches is 17. Collaboration between authors with higher publications and authors with one publication is commonly, but few collaborations were observed among the core authors. Additionally, the most influential authors formed a specific research teams, some teams focused on relatively similar research topic. This phenomenon reflects the collaboration among different teams in the future will be possible.

### Keyword Network Analysis

**Figure 5:**
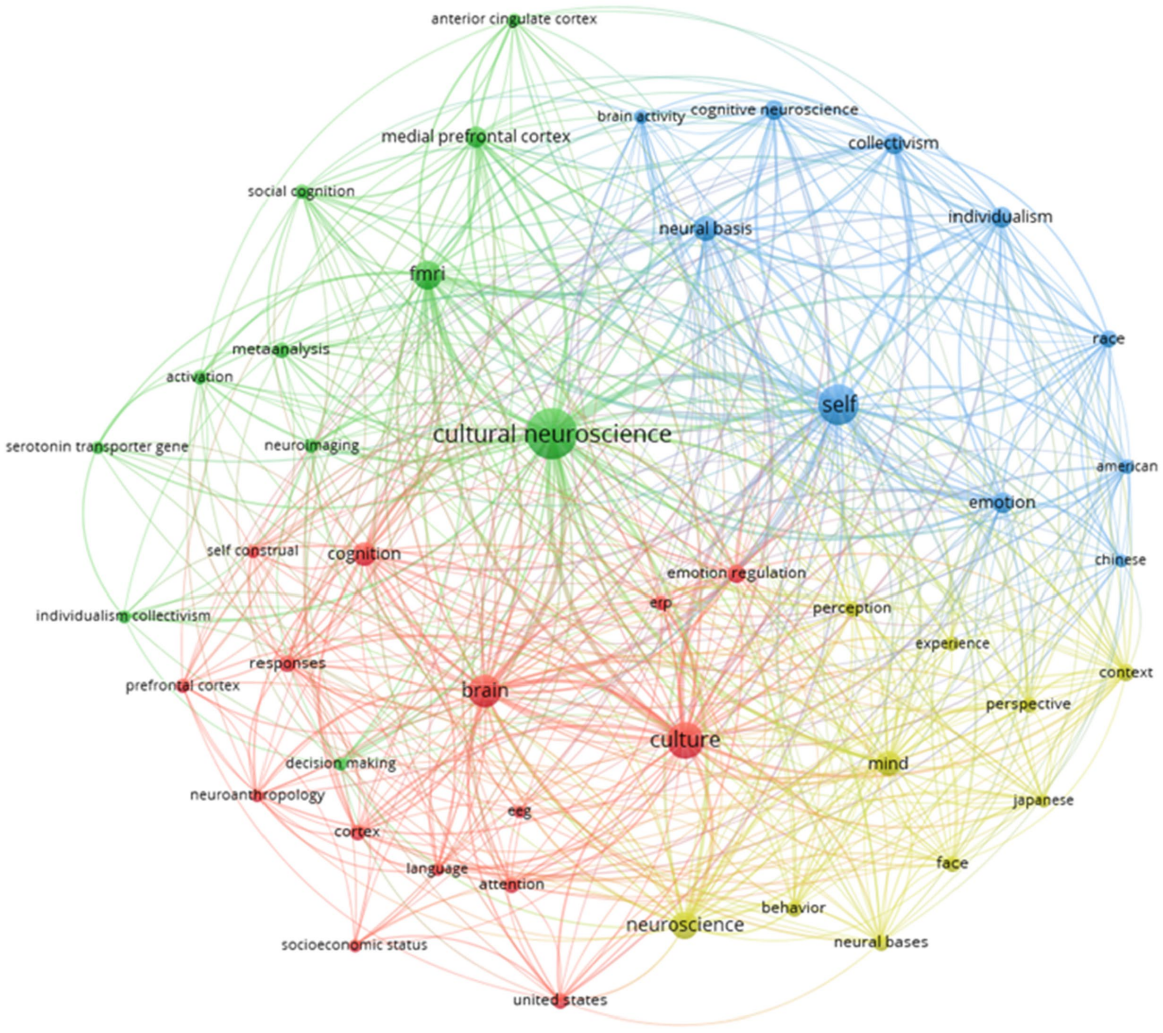
Keyword network.

The original keyword network was extracted hierarchically with a correlation frequency greater than or equal to five. Figure 5 shows the final keyword network. There were 46 core keyword nodes and 553 connections between keywords, with a network clustering of 0.49. The keyword network in the field of cultural neuroscience showed a high clustering coefficient and small-world feature attributes (Watts and Strogatz, 1998). From the figure, it can be observed that cultural neuroscience (de = 44, oc = 72), culture (de = 44, co = 38), self (de = 42, oc = 46), brain (d = 42, oc = 30), fmri (de = 38, oc = 23) are at the core of the keyword network and the key research points of scholars.

### Bibliographic Coupling Network Analysis

**Figure 6:**
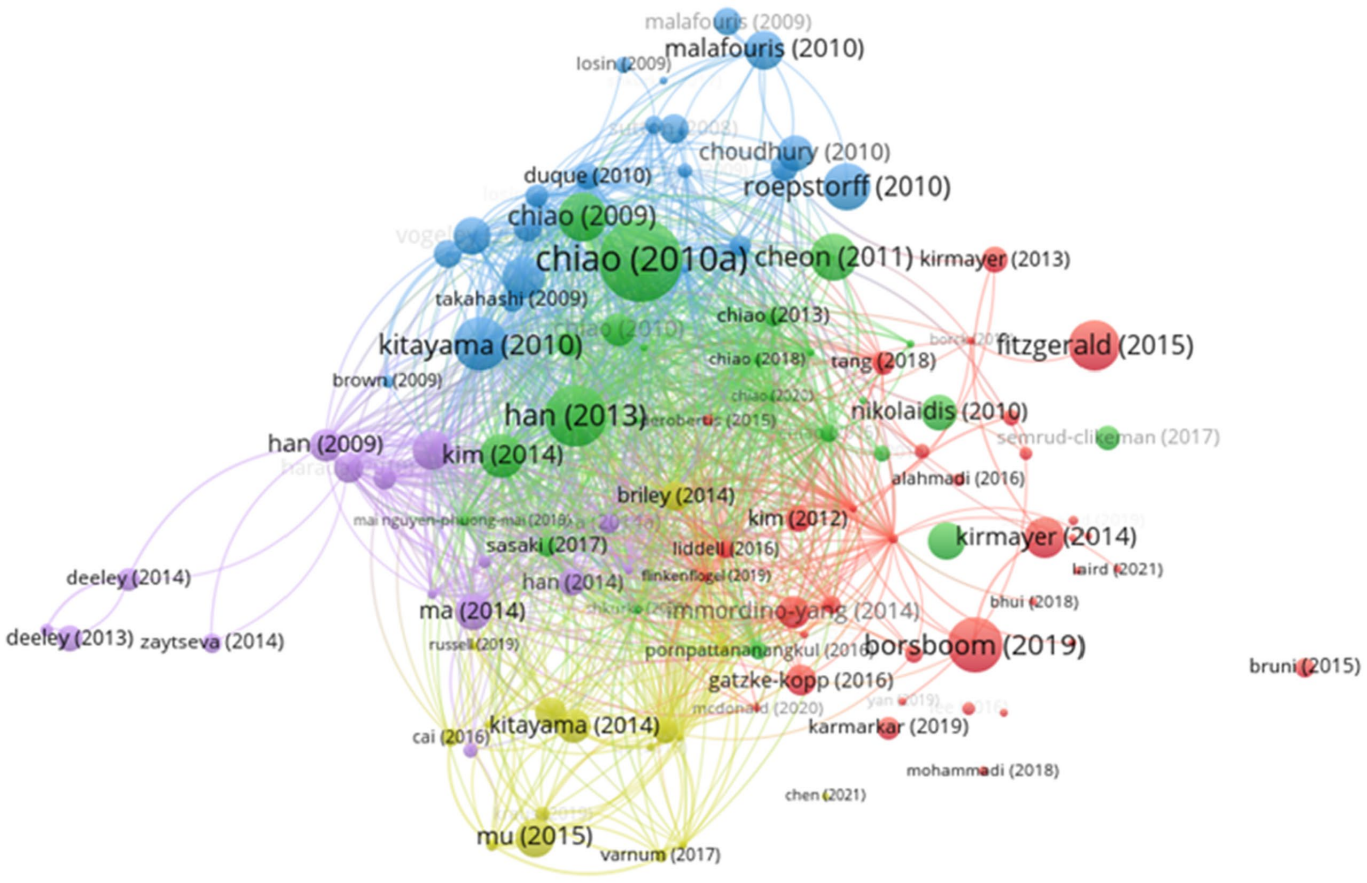
Bibliographic coupling network.

Keyword networks quickly identify Hot topics In a field. However, this method could only describe Hot topics In cultural neuroscience at a macro level, which Was compensated for By bibliographic coupling analysis (Kessler, 1963). Figure 6 shows the bibliographic coupling network formed by papers published in the field of cultural neuroscience in the period 2008–2021. A total of 113 papers have generated six major knowledge community clusters, and only five clusters contained >10 papers. Five cluster communities contained 112 documents, simultaneously forming The largest connected component. Table 7 presents data related to cluster topics.

**Table 7:** Bibliographic coupling network clustering data.

Year			Cluster		
	#1	#2	#3	#4	#5
2008			1		
2009		1	8		1
2010		3	8		1
2011		2			
2012	1		1		2
2013	2	3			1
2014	3	3	2	3	5
2015	4	2		1	
2016	6	1		1	1
2017	3	5	2	2	3
2018	5	1		1	1
2019	5	1		3	
2020	3	2			
2021	3			5	
TP	35	24	22	16	15
TC	642	1,007	777	256	374

*TP represents the total number of papers under a cluster knowledge community and TC represents the total number of citations for all articles under a cluster knowledge community*.

Table 7 shows the distribution of literature data over the years. Specifically #1 and #2 represented the knowledge communities with the highest numbers of articles and citations, respectively. From the perspective of evolutionary characteristics knowledge communities #1 and #2 have published the most papers in the past three years. However knowledge clusters #3 and #5 have not published studies since 2019. Therefore knowledge clusters #1 and #4 could possibly be the future research trends in cultural neuroscience while #3 and #5 might have faded out of the research perspectives of scholars. We further conducted an in-depth analysis of the literature contained in the five knowledge communities as given below.

Cultural Neuroscience and Neuroimaging, Psychiatric Diseases #1: This was the largest knowledge community in the field of cultural neuroscience, with 35 articles and 642 citations. Research in this area has primarily focused on the mechanisms of action in cultural neuroscience for analyzing neuroimaging and psychiatric diseases. For example, Borsboom et al. (2019) proposed the use of network models as a surrogate framework for analyzing mental disorders and found that cultural and historical backgrounds largely affected the strength of network relationships. Kirmayer and Ban (2013) explored the influence of cultural neuroscience on cultural psychiatry research theory and practice. More specific research questions included cultural variations in illness experience and expression, cultural configurations of self and personhood, and the prospect of ecosocial models of health and culturally based interventions, among others. Crafa and Nagel (2020) advocated the adoption of the culture–brain–behavior (CBB) interaction model for conceptualizing the relationship between culture, brain, and psychiatric disorders, stating that the integration of cultural neuroscience methods into cross-cultural psychiatry research could effectively address the individuality and cultural diversity of patients’ sexual influence. Immordino-Yang et al. (2014) used fMRI and electrocardiogram (ECG) techniques, along with observations of participants in China and the United States, to discover how Al activity correlated with feelings in social emotions and whether this correlation might be affected by cultural influence. The findings suggested that the brain’s ability to construct social–emotional conscious experiences was partly influenced by culture.

Cultural Neuroscience and Theoretical Methods #2: This was the most cited knowledge community, containing 24 articles and 1,007 citations. Scholars in this knowledge community focused on the theoretical methods and developmental trends of cultural neuroscience. Among them, Chiao (2009) introduced the research purposes and methods of cultural neuroscience and discussed the potential implications of this field for bridging the social and natural sciences, in addition to addressing the interethnic ideology and population health concern. A series of core theoretical and methodological challenges faced by researchers in conducting cultural neuroscience research is summarized and recommendations are provided for overcoming these challenges, eventually discussing the impact of cultural neuroscience research on addressing current health disparities in populations (Chiao et al., 2010). “Annual Review of Psychology,” the top-ranked journal in psychology, published an article in 2013, describing the origins, goals, methods, conceptual framework, and key findings of cultural neuroscience. The impact of cultural neuroscience research efforts on understanding human brain function was also discussed in sociocultural contexts, in addition to the emerging questions that should be addressed in future cultural neurological research (Han et al., 2013). The status of cultural neuroscience research and problems to be resolved were reviewed in 2014 (Kim and Sasaki, 2014).

Cultural Neuroscience and Interdisciplinary #3: The third largest knowledge cluster contained 22 articles and 777 citations. These articles primarily explored the application of cultural neuroscience in different disciplines. For example, Brown and Seligman (2009) provided constructive suggestions for the intersection of cultural neuroscience and anthropology, suggesting that anthropology could help locate unique or interesting populations and phenomena for cultural neuroscience research. Choudhury (2010) reapplied cultural neuroscience methods to anthropology to explore the characteristics of cultural change in the adolescent brain. Subsequent research combined cultural neuroscience with archaeology (Malafouris, 2009, 2010), neuroanthropology (Duque et al., 2010; Shkurko, 2017), and mathematics (Tcheang, 2014), and so on. Thus, scholars have persistently explored newly derived research themes under the cross-integration of different disciplines.

Cultural Neuroscience and Cultural Differences #4: The fourth largest cluster contained 16 articles with 256 citations. These articles mainly explored the differences in the social self of different cultures (collectivism and individualism), especially Asia, Europe, and America. Kitayama and Park (2014) compared the impact of culture on error-related negativity (ERN) egocentric effect by assessing cortical electrical responses in Europeans and Asians while performing flanking tasks. In the same year, the experimental study was launched again, finding that ERN could be used as an empirical marker of self-threat and was closely regulated by sociocultural variables (Park and Kitayama, 2014). Mu et al. (2015) employed electroencephalogram (EEG) in conjunction with a new social norm violation paradigm to examine the neural mechanisms that detected norm violations and identify how they differed across cultures (United States, China). The findings suggested that cultural differences had unique implications for social norm violation detection. Kraus et al. (2021) complemented the missing link in cultural psychology self-perception by comparing resting-state alpha strength (RSAP) and self-construct (SC) data relationships in European-Americans, Taiwanese, and Japanese. Hampton et al. (2021) found the influence of cultural differences on the ability to regulate emotional neural responses by testing samples from three different cultural groups: European-Americans, Mexicans, and Chinese.

Cultural Neuroscience and Brain Function #5: The fifth largest knowledge cluster contained 15 articles with 374 citations. These articles mainly provided support for cultural neuroscience research on brain function. For instance, Sul et al. (2012) found that the medial prefrontal cortex, anterior cingulate, bilateral temporoparietal, and precuneus were involved in the self-representation of personality traits and social identity, suggesting that a person’s cultural orientation can influence the brain’s self-reference effect (SRE). Ma et al. (2014b) explored how culturally specific self-construals were mediated by the human brain, using fMRI techniques to observe adults from East Asian (China) and Western (Denmark) cultural backgrounds. They found that individuals in different sociocultural contexts might learn and/or adopt distinct strategies for self-reflection by changing the weight of the medial prefrontal cortex (mPFC) and temporoparietal junction (TPJ) in the social brain network. Finally, Pfeifer et al. (2017) conducted an experiment on 14 Chinese youth to determine the role of TPJ in the self-evaluation process.

### Research Frontier Analysis

**Figure 7:**
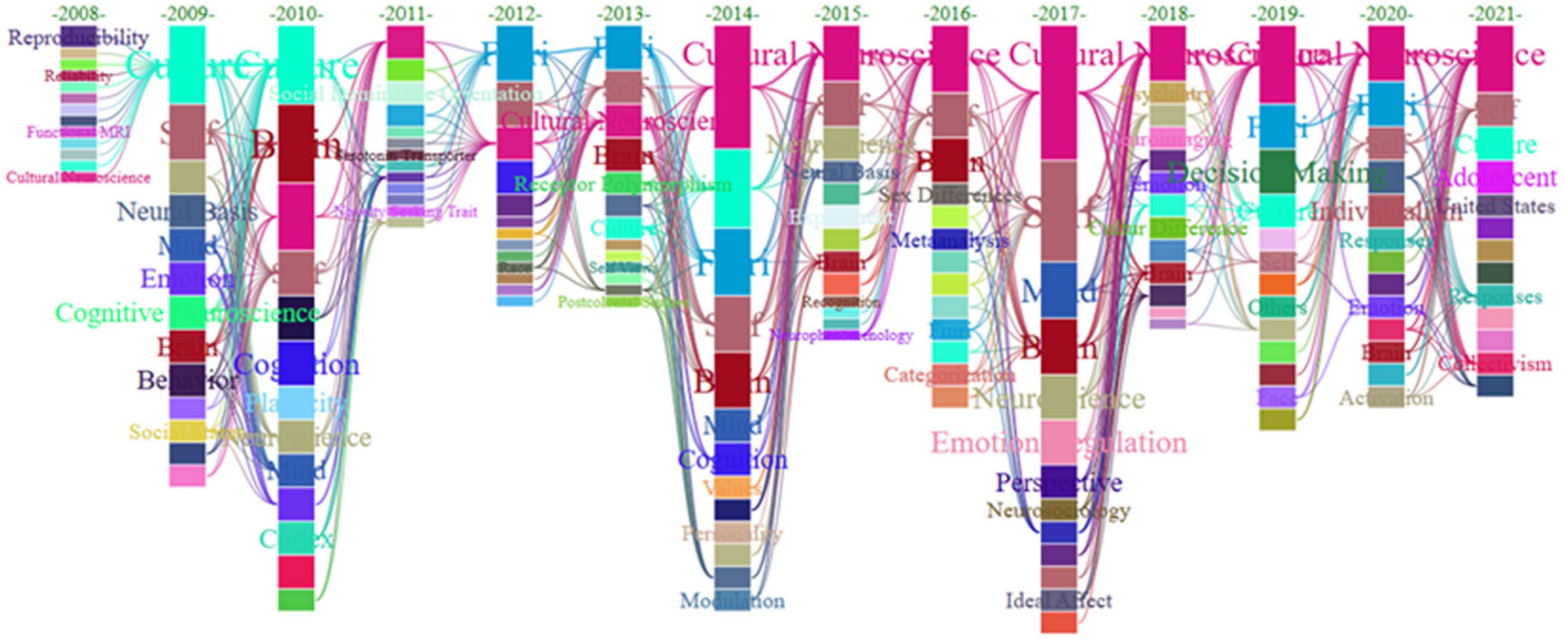
Theme evolution trend.

The static keyword network could not reveal the latest developmental trends in the field of cultural neuroscience research. Therefore, we analyzed the evolution characteristics of popular keywords within the time window of the past few years, using a time series analysis. Figure 7 shows the results of analyzing the evolution characteristics of keywords. The keyword nodes are sorted according to the frequency of occurrence; the higher the frequency, the closer the ranking is to the top. The connection between keywords in adjacent time periods indicated the relevance and continuity between keywords. Clearly, cultural neuroscience has gradually emerged as the hottest research topic since 2014. At the technical level, fMRI has been widely used by scholars since 2019 (Flinkenflogel et al., 2019; Murray et al., 2020; Shkurko, 2020), culture and self have remained the focus of researchers (Russell et al., 2019; Chiao et al., 2020; Kwon et al., 2021), and adolescent has emerged as the intellectual theme in 2021 (Chen and Qu, 2021; Rapp et al., 2021). Thus, the use of fMRI technology, combined with cultural neuroscience methods to study the impact of Chinese and Western cultural differences on brain function, appeared to be the future research trend.

## Conclusion and Contributions

### Conclusion and Discussion

This research analyzed 113 relevant documents in the field of “cultural neuroscience” using data from the core collection of Web of Science. Performance, collaboration network, keyword network, literature coupling network, and evolution analyses were performed to examine the evolution and development of the field of “cultural neuroscience.” The research conclusions were as follows:

The H-index of research papers in the field of cultural neuroscience was 32, indicating that this research discipline has received considerable attention from academia. Through performance analysis, we found that the United States occupied the most prominent position in cultural neuroscience research. Specifically, it was the most productive country, with Northwestern University, University of Michigan, and Peking University as the most influential research institutions, Chiao Jy as the most influential scholar, and “Social Cognitive and Affective Neuroscience” as the most influential journal. This would help scholars engaged in cultural neuroscience research better understand the development of research in the field.

The collaboration network analysis showed that the characteristics of collaboration in the field of cultural neuroscience have evolved from fragmentation to huge branches. As interdisciplinary fields of study have greater long-term effects than single-disciplinary studies (Van Noorden, 2015), research on interdisciplinary collaboration in the field of cultural neuroscience should be the future research direction.

The keyword network and literature coupling network analyses revealed that the knowledge network in the field of cultural neuroscience had a high clustering coefficient, with the characteristics of a small-world network, and has developed into a mature and stable subject field. The main research directions included a combination of cultural neuroscience with neuroimaging and mental disorders, theoretical methods, interdisciplinary integration, cultural differences (collectivism and individualism), and cultural neuroscience and brain function. Among them, neuroimaging, mental illness, and cultural differences (collectivism and individualism) remained the focus of future research by scholars.

The evolution analysis of time series showed that culture neuroscience, and self and culture, were the research focus and future research direction in the field of cultural neuroscience, respectively, while adolescent was the emerging research frontier.

### Future Directions

This research aims to encourage the application of cultural neuroscience in various disciplines, especially social sciences. First, fMRI technology can help us better perform the related tasks of cultural neuroscience research, but the complexity and cost of using this technology cannot be ignored. Therefore, future research work must determine ways to optimize the usage cost of this technology and realize the application value of fMRI in studying cultural neuroscience.

The results showed that researchers mostly compared the effects of cultural differences, in terms of East–West differences. Therefore, it is necessary to systematically explore other races and consider cultural diversity, especially ethnic groups with different cultural backgrounds in the same region. Understanding cross-cultural differences and conflicts (Chin et al., 2022) could help explore a missing link in theorizing contemporary cultural psychology (Kraus et al., 2021). Therefore, cross-cultural comparisons might provide a good direction for conducting future research.

The COVID-19 pandemic had a huge impact on the mental health of individuals, and social isolation adversely affected both the physical and mental health of people (Pietrabissa et al., 2021). Additionally, COVID-19 has also caused immense psychological stress in people (Liu et al., 2021). The results of this study revealed that applying cultural neuroscience to the treatment of psychiatric disorders has become a research priority. Therefore, using cultural neuroscience methods for mental health treatment during the pandemic is expected to be a future research trend.

Furthermore, the application of cultural neuroscience methods to social science is limited, which hinders the rapid development of cultural neuroscience. Therefore, it is necessary to determine ways to conceptualize and explicate biology–social relations in practice, such as psychiatry research (Fletcher and Birk, 2022). Additionally, how different brain regions organize to construct a topological network for the representation of individual’s cultural tendency, remains unclear (Luo et al., 2022). Thus, future studies must construct broader biology–social theoretical methods and standard frameworks.

### Theoretical and Practical Contributions

This study used bibliometric analysis to provide a comprehensive review of the field of cultural neuroscience, assess the latest research trends, overcome subjective judgments, and create reproducible research results. However, cross-regional and cross-institutional scientific research cooperation was observed as the developmental trend of cultural neuroscience, which could be beneficial to scientific researchers in terms of “increasing the probability of successful publication,” “project funding,” “reference of theoretical ideas,” and so on. On the practical side, we presented the current developments in cultural neuroscience, uncovered fundamental questions, and provided constructive suggestions for future research directions. Specifically, we provided researchers with a research reference point, such as choosing appropriate journals for publication and identifying a suitable research direction.

### Limitations

The limitations of this study are as follows. Only the core collection of Web of Science databases was used for the analysis, implying that some important documents in other databases were excluded. Therefore, future research could adopt more databases, such as SCOPUS and EBSCO. In conclusion, this study provides comprehensive information for relevant researchers and institutions, in order to enable them to understand the developmental trends in cultural neuroscience and provide a reference point for conducting future research.

## Author Contributions

HX planned the study, collected the data, and wrote the manuscript. HX and CY analyzed the data and wrote the manuscript. C-CC mainly revised and edited the manuscript. All authors contributed to the article and approved the submitted version.

## Funding

This research was funded by College of Science and Technology Ningbo University (YK202129) and K. C. Wong Magna Fund in Ningbo University.

## Conflict of Interest

The authors declare that the research was conducted in the absence of any commercial or financial relationships that could be construed as a potential conflict of interest.

## Publisher’s Note

All claims expressed in this article are solely those of the authors and do not necessarily represent those of their affiliated organizations, or those of the publisher, the editors and the reviewers. Any product that may be evaluated in this article, or claim that may be made by its manufacturer, is not guaranteed or endorsed by the publisher.

## References

[ref1] Alhibshi A. H. Alamoudi W. A. Ul Haq I. Rehman S. U. Farooq R. K. Al Shamrani F. J. (2020). Bibliometric analysis of neurosciences research productivity in Saudi Arabia from 2013-2018. Neurosciences 25, 134–143. doi: 10.17712/nsj.2020.2.20190087, PMID: 32351251 PMC8015521

[ref2] Bai Y. Li H. X. Liu Y. (2021). Visualizing research trends and research theme evolution in E-learning field: 1999-2018. Scientometrics 126, 1389–1414. doi: 10.1007/s11192-020-03760-7

[ref3] Bhui K. (2018). Cultural neuroscience: ideas worth knowing. Nord. J. Psychiatry 72, S5–S8. doi: 10.1080/08039488.2018.1525647, PMID: 30380972

[ref4] Blondel V. D. Guillaume J. L. Lambiotte R. Lefebvre E. (2008). Fast unfolding of communities in large networks. J. Stat. Mech. Theory Exp. 2008:P10008. doi: 10.1088/1742-5468/2008/10/P10008

[ref5] Borsboom D. Cramer A. O. J. Kalis A. (2019). Brain disorders? Not really: why network structures block reductionism in psychopathology research. Behav. Brain Sci. 42:e2. doi: 10.1017/s0140525x17002266 29361992

[ref6] Broadus R. N. (1987). Toward a definition of “bibliometrics”. Scientometrics 12, 373–379. doi: 10.1007/BF02016680

[ref7] Brown R. A. Seligman R. (2009). “Anthropology and cultural neuroscience: creating productive intersections in parallel fields,” in Cultural Neuroscience: Cultural Influences on Brain Function. *Vol*. 178. ed. Chiao J. Y., 31–42. 10.1016/S0079-6123(09)17803-2 19874960

[ref8] Chen P. H. A. Qu Y. (2021). Taking a computational cultural neuroscience approach to study parent-child similarities in diverse cultural contexts. Front. Hum. Neurosci. 15:703999. doi: 10.3389/fnhum.2021.703999, PMID: 34512293 PMC8426574

[ref9] Chiao J. Y. (2009). “Cultural neuroscience: a once and future discipline,” in Cultural Neuroscience: Cultural Influences on Brain Function. *Vol*. 178. ed. Chiao J. Y. 287–304. 10.1016/S0079-6123(09)17821-4 19874977

[ref10] Chiao J. Y. (2018). Developmental aspects in cultural neuroscience. Dev. Rev. 50, 77–89. doi: 10.1016/j.dr.2018.06.005, PMID: 30778272 PMC6377197

[ref11] Chiao J. Y. Blizinsky K. D. (2010). Culture-gene coevolution of individualism-collectivism and the serotonin transporter gene. Proceed. Royal Soc. B-Bio. Sci 277, 529–537. doi: 10.1098/rspb.2009.1650, PMID: 19864286 PMC2842692

[ref12] Chiao J. Y. Hariri A. R. Harada T. Mano Y. Sadato N. Parrish T. B.. (2010). Theory and methods in cultural neuroscience. Soc. Cogn. Affect. Neurosci. 5, 356–361. doi: 10.1093/scan/nsq063, PMID: 20592044 PMC2894689

[ref13] Chiao J. Y. Li S. C. Turner R. Lee-Tauler S. Y. (2020). Cultural neuroscience and the research domain criteria: implications for global mental health. Neurosci. Biobehav. Rev. 116, 109–119. doi: 10.1016/j.neubiorev.2020.06.005, PMID: 32540352

[ref14] Chin T. C. Meng J. W. Wang S. Y. Shi Y. Zhang J. N. (2022). Cross-cultural metacognition as a prior for humanitarian knowledge: when cultures collide in global health emergencies. J. Knowl. Manag. 26, 88–101. doi: 10.1108/Jkm-10-2020-0787

[ref15] Choudhury S. (2010). Culturing the adolescent brain: what can neuroscience learn from anthropology? Soc. Cogn. Affect. Neurosci. 5, 159–167. doi: 10.1093/scan/nsp030, PMID: 19959484 PMC2894667

[ref16] Crafa D. Nagel S. K. (2020). Traces of culture: the feedback loop between behavior, brain, and disorder. Transcult. Psychiatry 57, 387–407. doi: 10.1177/1363461519879515, PMID: 31996101

[ref17] Donthu N. Kumar S. Mukherjee D. Pandey N. Lim W. M. (2021a). How to conduct a bibliometric analysis: an overview and guidelines. J. Bus. Res. 133, 285–296. doi: 10.1016/j.jbusres.2021.04.070

[ref18] Donthu N. Kumar S. Pandey N. Gupta P. (2021b). Forty years of the international journal of information management: a bibliometric analysis. Int. J. Inf. Manag. 57:102307. doi: 10.1016/j.ijinfomgt.2020.102307

[ref19] Duque J. F. D. Turner R. Lewis E. D. Egan G. (2010). Neuroanthropology: a humanistic science for the study of the culture-brain nexus. Soc. Cogn. Affect. Neurosci. 5, 138–147. doi: 10.1093/scan/nsp024, PMID: 19654141 PMC2894669

[ref20] Fatt C. K. Abu Ujum E. Ratnavelu K. (2010). The structure of collaboration in the journal of finance. Scientometrics 85, 849–860. doi: 10.1007/s11192-010-0254-0

[ref21] Fitzgerald D. Callard F. (2015). Social science and neuroscience beyond Interdisciplinarity: experimental entanglements. Theory Culture Soc 32, 3–32. doi: 10.1177/0263276414537319, PMID: 25972621 PMC4425296

[ref22] Fletcher J. R. Birk R. H. (2022). The conundrum of the psychological interface: On the problems of bridging the biological and the social. Hist. Hum. Sci. doi: 10.1177/09526951211070503

[ref23] Flinkenflogel N. Vu T. V. van Kesteren M. T. R. Krabbendam L. (2019). Neural correlates of self-construal priming in the ultimatum game. Front. Neurosci. 13:994. doi: 10.3389/fnins.2019.00994, PMID: 31616239 PMC6769036

[ref24] Gonzalez-Alcaide G. Park J. Huamani C. Belinchon I. Ramos J. M. (2015). Evolution of cooperation patterns in psoriasis research: co-authorship network analysis of papers in Medline (1942-2013). PLoS One 10:e0144837. doi: 10.1371/journal.pone.0144837, PMID: 26658481 PMC4676628

[ref25] Hamada T. (2018). Japanese Company's cultural shift for gender equality at work. Glob. Econ. Rev. 47, 63–87. doi: 10.1080/1226508x.2017.1393725

[ref26] Hampton R. S. Kwon J. Y. Varnum M. E. W. (2021). Variations in the regulation of affective neural responses Across three cultures. Emotion 21, 283–296. doi: 10.1037/emo0000711, PMID: 31815497

[ref27] Han S. H. Northoff G. Vogeley K. Wexler B. E. Kitayama S. Varnum M. E. W. (2013). “A cultural neuroscience approach to the biosocial nature of the human brain,” in Annual Review of Psychology. *Vol*. 64. ed. Fiske S. T., 335–359. 10.1146/annurev-psych-071112-054629 22994921

[ref29] Hosseini M. R. Martek I. Zavadskas E. K. Aibinu A. A. Arashpour M. Chileshe N. (2018). Critical evaluation of off-site construction research: a Scientometric analysis. Autom. Constr. 87, 235–247. doi: 10.1016/j.autcon.2017.12.002

[ref30] Immordino-Yang M. H. Yang X. F. Damasio H. (2014). Correlations between social-emotional feelings and anterior insula activity are independent from visceral states but influenced by culture. Front. Hum. Neurosci. 8:728. doi: 10.3389/fnhum.2014.00728, PMID: 25278862 PMC4165215

[ref31] Issac A. C. Issac T. G. (2020). Unravelling the nexus between neuroscience and leadership research A biblio-morphological analysis of the extant literature. Manag. Decis. 58, 448–464. doi: 10.1108/md-01-2019-0017

[ref32] Kessler M. M. (1963). Bibliographic coupling between scientific papers. J. Am. Soc. Inf. Sci. Technol. 14, 10–25.

[ref33] Khan K. I. Nasir A. Saleem S. (2021). Bibliometric analysis of post Covid-19 management strategies and policies in hospitality and tourism. Front. Psychol. 12:769760. doi: 10.3389/fpsyg.2021.769760, PMID: 34867674 PMC8634669

[ref34] Kim H. S. Sasaki J. Y. (2014). Cultural neuroscience: biology of the mind in cultural contexts. In S. T. Fiske (Ed.). Annu. Rev. Psychol. 65, 487–514. doi: 10.1146/annurev-psych-010213-115040, PMID: 24050186

[ref35] Kirmayer L. J. Ban L. (2013). “Cultural psychiatry: research strategies and future directions,” in Cultural Psychiatry. *Vol*. 33. ed. Alarcon R. D., 97–114. 10.1159/000348742 23816867

[ref36] Kitayama S. Park J. (2010). Cultural neuroscience of the self: understanding the social grounding of the brain. Soc. Cogn. Affect. Neurosci. 5, 111–129. doi: 10.1093/scan/nsq052, PMID: 20592042 PMC2894676

[ref37] Kitayama S. Park J. (2014). Error-related brain activity reveals self-centric motivation: culture matters. J. Exp. Psychol. General 143, 62–70. doi: 10.1037/a0031696, PMID: 23398181

[ref38] Kitayama S. Salvador C. E. (2017). Culture Embrained: going Beyond the nature-nurture dichotomy. Perspect. Psychol. Sci. 12, 841–854. doi: 10.1177/1745691617707317, PMID: 28972851 PMC5841951

[ref39] Kraus B. Salvador C. E. Kamikubo A. Hsiao N. C. Hu J. F. Karasawa M.. (2021). Oscillatory alpha power at rest reveals an independent self: a cross-cultural investigation. Biol. Psychol. 163:108118. doi: 10.1016/j.biopsycho.2021.108118, PMID: 34019966 PMC8491569

[ref40] Kwon J. Y. Wormley A. S. Varnum M. E. W. (2021). Changing cultures, changing brains: a framework for integrating cultural neuroscience and cultural change research. Biol. Psychol. 162:108087. doi: 10.1016/j.biopsycho.2021.108087, PMID: 33781802

[ref41] Liu P. Wang X. F. Li D. Zhang R. W. Li H. Han J. X. (2021). The benefits of self-transcendence: examining the role of values on mental health Among adolescents Across regions in China. Front. Psychol. 12:630420. doi: ARTN 630420 10.3389/fpsyg.2021.630420 33679555 PMC7925830

[ref42] Lozano S. Calzada-Infante L. Adenso-Diaz B. Garcia S. (2019). Complex network analysis of keywords co-occurrence in the recent efficiency analysis literature. Scientometrics 120, 609–629. doi: 10.1007/s11192-019-03132-w

[ref43] Luo H. F. Cai Z. L. Huang Y. Y. Song J. T. Ma Q. Yang X. W.. (2021). Study on pain Catastrophizing From 2010 to 2020: a Bibliometric analysis via CiteSpace. Front. Psychol. 12:759347. doi: 10.3389/fpsyg.2021.759347, PMID: 34975649 PMC8718514

[ref44] Luo S. Y. Zhu Y. Y. Han S. H. (2022). Functional connectome fingerprint of holistic-analytic cultural style. Soc. Cogn. Affect. Neurosci. 17, 172–186. doi: 10.1093/scan/nsab080, PMID: 34160613 PMC8847908

[ref45] Ma Y. N. Bang D. Wang C. B. Allen M. Frith C. Roepstorff A.. (2014a). Sociocultural patterning of neural activity during self-reflection. Soc. Cogn. Affect. Neurosci. 9, 73–80. doi: 10.1093/scan/nss103, PMID: 22956678 PMC3871729

[ref46] Ma Y. N. Wang C. B. Li B. F. Zhang W. X. Rao Y. Han S. H. (2014b). Does self-construal predict activity in the social brain network? A genetic moderation effect. Soc. Cogn. Affect. Neurosci. 9, 1360–1367. doi: 10.1093/scan/nst125, PMID: 24009354 PMC4158375

[ref47] Malafouris L. (2009). “Neuroarchaeology: exploring the links between neural and cultural plasticity,” in Cultural Neuroscience: Cultural Influences on Brain Function. *Vol*. 178. ed. Chiao J. Y., 253–261. 10.1016/S0079-6123(09)17818-4 19874975

[ref48] Malafouris L. (2010). The brain-artefact interface (BAI): a challenge for archaeology and cultural neuroscience. Soc. Cogn. Affect. Neurosci. 5, 264–273. doi: 10.1093/scan/nsp057, PMID: 20123661 PMC2894672

[ref49] Marques I. C. P. Franco M. (2020). Cooperation networks in the area of health: systematic literature review. Scientometrics 122, 1727–1750. doi: 10.1007/s11192-019-03341-3

[ref50] McAuley J. J. Costa L. D. F. Caetano T. S. (2007). Rich-club phenomenon across complex network hierarchies. Appl. Phys. Lett. 91:084103. doi: 10.1063/1.2773951

[ref51] Mu Y. Kitayama S. Han S. H. Gelfand M. J. (2015). How culture gets embrained: cultural differences in event-related potentials of social norm violations. Proc. Natl. Acad. Sci. U. S. A. 112, 15348–15353. doi: 10.1073/pnas.1509839112, PMID: 26621713 PMC4687606

[ref52] Murray L. Lopez-Duran N. L. Mitchell C. Monk C. S. Hyde L. W. (2020). Neural mechanisms of reward and loss processing in a low-income sample of at-risk adolescents. Soc. Cogn. Affect. Neurosci. 15, 1299–1314. doi: 10.1093/scan/nsaa157, PMID: 33216937 PMC7759206

[ref53] Nguyen T. N. Abdalkader M. Jovin T. G. Nogueira R. G. Jadhav A. P. Haussen D. C.. (2020). Mechanical Thrombectomy in the era of the COVID-19 pandemic: emergency preparedness for neuroscience teams A guidance statement From the Society of Vascular and Interventional Neurology. Stroke 51, 1896–1901. doi: 10.1161/strokeaha.120.030100, PMID: 32347790 PMC7202095

[ref54] Novo N. R. Munoz M. M. N. Cuellar-Pompa L. Gomez J. A. R. (2021). Trends in research on art therapy indexed in the web of science: a Bibliometric analysis. Front. Psychol. 12:752026. doi: 10.3389/fpsyg.2021.752026, PMID: 34867642 PMC8639497

[ref55] Park J. Kitayama S. (2014). Interdependent selves show face-induced facilitation of error processing: cultural neuroscience of self-threat. Soc. Cogn. Affect. Neurosci. 9, 201–208. doi: 10.1093/scan/nss125, PMID: 23160814 PMC3907928

[ref56] Pfeifer J. H. Mahy C. E. V. Merchant J. S. Chen C. H. Masten C. L. Fuligni A. J.. (2017). Neural Systems for Reflected and Direct Self-Appraisals in Chinese young adults: exploring the role of the temporal-parietal junction. Cultur. Divers. Ethnic Minor. Psychol. 23, 45–58. doi: 10.1037/cdp0000122, PMID: 28045310 PMC10826844

[ref57] Pietrabissa G. Volpi C. Bottacchi M. Bertuzzi V. Usubini A. G. Loffler-Stastka H.. (2021). The impact of social isolation during the COVID-19 pandemic on physical and mental health: The lived experience of adolescents with obesity and their caregivers. Int. J. Environ. Res. Public Health 18:3026. doi: 10.3390/ijerph18063026, PMID: 33804231 PMC7999166

[ref58] Rapp A. M. Grammer J. K. Tan P. Z. Gehring W. J. Chavira D. A. Miller G. A. (2021). Collectivism is associated with enhanced neural response to socially salient errors among adolescents. Soc. Cogn. Affect. Neurosci. 16, 1150–1159. doi: 10.1093/scan/nsab065, PMID: 34041547 PMC8599179

[ref59] Russell M. J. Masuda T. Hioki K. Singhal A. (2019). Culture and neuroscience: how Japanese and European Canadians process social context in close and acquaintance relationships. Soc. Neurosci. 14, 484–498. doi: 10.1080/17470919.2018.1511471, PMID: 30103645

[ref60] Shi Z. H. Ma Y. N. Wu B. Wu X. H. Wang Y. Y. Han S. H. (2016). Neural correlates of reflection on actual versus ideal self-discrepancy. NeuroImage 124, 573–580. doi: 10.1016/j.neuroimage.2015.08.077, PMID: 26375210

[ref61] Shkurko Y. S. (2017). In search of neurosociology. Sotsiologicheskie Issledovaniya 8, 4–11. doi: 10.7868/s0132162517080013

[ref62] Shkurko A. (2020). Mapping cultural values onto the brain: the fragmented landscape. Integr. Psychol. Behav. Sci. doi: 10.1007/s12124-020-09553-0, PMID: 32495163

[ref63] Sul S. Choi I. Kang P. (2012). Cultural modulation of self-referential brain activity for personality traits and social identities. Soc. Neurosci. 7, 280–291. doi: 10.1080/17470919.2011.614001, PMID: 21970690

[ref64] Sutton B. P. Goh J. Hebrank A. Welsh R. C. Chee M. W. L. Park D. C. (2008). Investigation and validation of intersite fMRI studies using the same imaging hardware. J. Magn. Reson. Imaging 28, 21–28. doi: 10.1002/jmri.21419, PMID: 18581342 PMC2785504

[ref65] Tcheang L. (2014). Culture and math. Cogn. Neurosci. 5, 54–65. doi: 10.1080/17588928.2013.838552 24090438

[ref66] Vaiana M. Muldoon S. F. (2020). Multilayer brain networks. J. Nonlinear Sci 30, 2147–2169. doi: 10.1007/s00332-017-9436-8

[ref67] van Eck N. J. Waltman L. (2010). Software survey: VOSviewer, a computer program for bibliometric mapping. Scientometrics 84, 523–538. doi: 10.1007/s11192-009-0146-3, PMID: 20585380 PMC2883932

[ref68] Van Noorden R. (2015). Interdisciplinary research by the numbers. Nature 525, 306–307. doi: 10.1038/525306a 26381967

[ref69] Wang X. F. Zhang S. Liu Y. Q. (2021). ITGInsight-discovering and visualizing research fronts in the scientific literature. Scientometrics. doi: 10.1007/s11192-021-04190-9

[ref70] Wang Z. H. Zhao Y. D. Wang B. (2018). A bibliometric analysis of climate change adaptation based on massive research literature data. J. Clean. Prod. 199, 1072–1082. doi: 10.1016/j.jclepro.2018.06.183

[ref71] Watts D. J. Strogatz S. H. (1998). Collective dynamics of 'small-world' networks. Nature 393, 440–442. doi: 10.1038/30918 9623998

[ref72] Yan W. T. Lu S. Yang Y. D. Ning W. Y. Cai Y. Hu X. M. (2021). Research trends, hot spots and prospects for necroptosis in the field of neuroscience. Neural Regen. Res. 16, 1628–1637. doi: 10.4103/1673-5374.303032, PMID: 33433494 PMC8323674

[ref73] Yeung A. W. K. Goto T. K. Leung W. K. (2017). The changing landscape of neuroscience research, 2006-2015: a Bibliometric study. Front. Neurosci. 11:120. doi: 10.3389/fnins.2017.00120, PMID: 28377687 PMC5360093

